# Impact of comorbid depression on quality of life and disease progression in chronic obstructive pulmonary disease: a correlational analysis

**DOI:** 10.3389/fpsyt.2025.1667902

**Published:** 2025-10-08

**Authors:** Yu Chen, Qianqian Gao, Hongqin Jia, Kang Qian, Hongbin Zhu

**Affiliations:** ^1^ The Fourth Affiliated Hospital of Anhui Medical University, Chaohu, China; ^2^ Department of Respiratory and Critical Care Medicine, Fuyang People's Hospital, Fuyang, China; ^3^ Department of Respiratory and Critical Care Medicine, The Fourth Affiliate Hospital of Anhui Medical University, Chaohu, China

**Keywords:** AECOPD, depression, risk factors, quality of life, stigma

## Abstract

**Introduction:**

Depression is a prevalent comorbidity in patients with Chronic Obstructive Pulmonary Disease (COPD), particularly during acute exacerbations (AECOPD), significantly impacting prognosis and quality of life. This study aimed to investigate the risk factors and severity of depression in this population.

**Objective:**

To identify risk factors and assess depression severity in patients experiencing Acute Exacerbation of Chronic Obstructive Pulmonary Disease (AECOPD).

**Methods:**

AECOPD patients admitted to Chaohu Hospital Affiliated with Anhui Medical University, between October 2023 and February 2025 were included. Participants were divided into four groups using the Patient Health Questionnaire-9 (PHQ-9): mild depression group (n=86), moderate depression group (n=24), severe depression group (n=3), and a control group without depression (n=156). Data collection involved: the first two domains of the World Health Organization Quality of Life questionnaire (WHOQOL-BREF), The Social Impact Scale (SIS) for stigma assessment Clinical data, treatment history, and laboratory test results. A custom-designed questionnaire was utilized to record hospitalization details for intergroup comparisons.

**Results:**

This study included 269 AECOPD patients, comprising 113 cases in the depression group and 156 controls. Comparative analysis revealed that female patients, those with longer smoking histories, theophyllines users, individuals with greater disease severity, stronger perceived stigma, and poorer quality of life demonstrated higher susceptibility to depression. In depression severity subgroups, 86 cases were classified as mild depression while 27 cases exhibited moderate-to-severe depression. The results demonstrated that gender, glucocorticoid use, daily cigarette consumption, and prolonged hospitalization were significantly associated with aggravated comorbid depression in COPD patients.

**Conclusion:**

Depressive state in patients with AECOPD is clinically common and associated with factors including gender, smoking history, MMRC grade, disease severity, hospitalization duration, theophyllines use, as well as quality of life and stigma.

## 1 Introduction

Chronic obstructive pulmonary disease (COPD) is a prevalent yet preventable and treatable condition characterized by persistent respiratory symptoms and airflow limitation, primarily caused by prolonged exposure to noxious particles or gases like tobacco smoke (1). A major global health challenge, COPD ranks third leading cause of death and seventh highest health risk worldwide (2), with a 2022 age-standardized mortality rate of 10.44 per 100,000 (3).

Depression, a psychiatric disorder, features persistent depressed mood, anhedonia, and associated symptoms like psychomotor changes, fatigue, and impaired concentration (4). It also presents vegetative symptoms and exhibits high comorbidity with medical/psychiatric disorders, significant prevalence, and elevated recurrence/disability risks (5).

COPD frequently coexists with depression (6, 7), often underdiagnosed due to prioritization of respiratory symptoms (8, 9). GOLD emphasizes integrating depression screening. Prevalence is 10–42% in stable COPD and 10–86% during exacerbations (10), with up to 55% exhibiting psychiatric comorbidities (11). Major depressive disorder predominates. Depression interacts synergistically with modifiable risk factors like tobacco use, amplifying mortality (12).

Despite rising prevalence, depression’s risk factors in COPD remain poorly characterized (13). The specific role of inflammatory markers, oxidative stress parameters, and other objective laboratory data in conjunction pharmacological treatments and psychosocial measures during an exacerbation is poorly characterized. Our preliminary study found significant insomnia and depressive symptom comorbidity in AECOPD (14), prompting this investigation into determinants of depression. This study systematically examines associations between depressive states and laboratory biomarkers, medical history, and pharmacological interventions to inform targeted therapies.

## 2 Materials and methods

### 2.1 Study population

A total of 269 patients with Acute Exacerbation of Chronic Obstructive Pulmonary Disease (AECOPD), admitted to Chaohu Hospital Affiliated with Anhui Medical University between October 2023 and February 2025, were included in this study. Inclusion Criteria:(1) Diagnosis of COPD confirmed according to the 2023 Global Initiative for Chronic Obstructive Lung Disease (GOLD) diagnostic criteria. (2) Current hospitalization due to acute exacerbation, defined by rapid clinical deterioration requiring therapeutic modifications. Exclusion Criteria: (1) History of diagnosed psychiatric disorders, mental illness, or severe organ failure. (2) Use of antidepressants or sedatives within the past month. (3) Impaired consciousness or language dysfunction. (4) Visual or auditory impairments hindering questionnaire completion. The study protocol adhered to the principles of the Declaration of Helsinki and was approved by the Ethics Committee of Chaohu Hospital Affiliated with Anhui Medical University(Approval No.KYXM-202309-005). Written informed consent was obtained from all participants.

### 2.2 Data collection

(1) Baseline demographic data were collected through structured interviews, including age, sex, BMI, smoking history, widowhood status, educational level, comorbidities, mMRC dyspnea grade, and scores from depression, quality of life (QOL), and stigma assessments. (2) Clinical treatment data were extracted from medical records: annual hospitalization frequency, antibiotic types, theophyllines use, glucocorticoid administration methods, and current hospitalization duration. (3) Laboratory parameters from admission tests were recorded: complete blood count, hepatic/renal function, and coagulation panel.

### 2.3 Measurement tools

(1) Quality of Life (QOL): Evaluated using the first two domains of the World Health Organization Quality of Life Brief Version (WHOQOL-BREF), reflecting patients’ perceived health and life satisfaction. Higher total scores indicate better QOL. (2) Patient Health Questionnaire-9 (PHQ-9): Assessed depression severity over a 2-week period. Total scores range from 0–27 (each item scored 0–3):0–4: No/minimal depression, 5–9: Mild depression, 10–14: Moderate depression, 15–19: Moderately severe depression, ≥20: Severe depression; (3) Social Impact Scale (SIS): Measured perceived stigma using a 4-point Likert scale (1=“strongly disagree” to 4=“strongly agree”). Higher scores indicate greater stigmatization. (4) Modified Medical Research Council (mMRC) Dyspnea Scale: Graded COPD-related breathlessness:Grade 0: Dyspnea only during strenuous exercise, Grade 1: Shortness of breath when walking briskly or climbing slopes, Grade 2: Slower walking pace than peers due to breathlessness, Grade 3: Requires rest after walking 100 meters, Grade 4: Breathlessness at rest or during basic activities.

### 2.4 Statistical analysis

Data were analyzed using SPSS 27.0. This was an observational cross-sectional study without *a priori* sample size calculation. During the study period, 269 eligible patients with AECOPD were consecutively enrolled. A *post-hoc* power analysis indicated that, with quality of life (QOL) as the primary outcome, the observed between-group effect size was Cohen’s d≈1.36. Continuous variables with normal distribution were expressed as mean ± SD; non-normal data as median (IQR); categorical variables as n (%). Intergroup comparisons employed: Independent t-tests (normally distributed data with equal variances), Mann-Whitney U tests (non-normal/heteroscedastic data), Chi-square tests (categorical variables), Statistical significance was set at *p* < 0.05 (two-tailed).

## 3 Results

### 3.1 Clinical characteristics of AECOPD patients

The study included 269 patients, stratified into a depression group (n=113) and a control group (n=156) based on PHQ-9 scores. Significant differences (*p* < 0.05) were observed between groups in sex, smoking history, mMRC grades, stigma scores (SIS), and QOL scores. Specifically, the depression group exhibited a higher proportion of female patients, elevated smoking history, greater disease severity (higher mMRC grades), stronger perceived stigma, and poorer quality of life compared to controls. No statistically significant differences (*p*≥0.05) were found in age, BMI, marital status, comorbidities, educational attainment, hospitalization frequency, or length of hospital stay between groups (**Table 1**).

**Table 1:** The depression group demonstrated a significantly higher proportion of female patients compared to controls (*p* < 0.05).

Variable	Category	Control group (n = 156)	Depression group (n = 113)	Z/χ²/t	P-value
Age (years)		75(71~82)	76(71.5~80.5)	-0.218	0.828
Length of hospital stay (days)		8(7~10)	8(7.0~10.5)	0.343	0.731
Hospitalizations within past year (times)		1(1~2)	1(1~2)	1.299	0.194
Sex	Male	127(81.40%)	80(70.80%)	4.162	0.041
	Female	29(18.60%)	33(29.20%)		
BMI		20.988 ± 4.073	21.123 ± 3.364	-0.297	0.767
Education level	Primary school or below	132(84.60%)	93(82.30%)	0.257	0.612
	Above primary school	24(15.40%)	20(17.70%)		
Smoking history (years)		40(0~50)	30(0~40)	-1.910	0.056
Comorbidities	No	45(28.80%)	38(33.60%)	0.702	0.402
	Yes	111(71.20%)	75(66.40%)		
Daily cigarette consumption (sticks)		20(0~40)	20(0~40)	-0.547	0.584
mMRCgrade	Grade 0	0(0.00%)	0(0.00%)	43.151	<0.001
	Grade 1	42(26.90%)	6(5.30%)		
	Grade 2	37(23.70%)	20(17.70%)		
	Grade 3	42(26.90%)	22(19.50%)		
	Grade 4	35(22.40%)	65(57.50%)		
SIS		51.000(49.000~53.750)	54(47.5~56)	3.297	<0.001
QOL		56.081 ± 4.22724	50.5729 ± 3.89135	10.903	<0.001

Marked distributional disparities were observed in mMRC grades between groups (*p* < 0.001). Patients in the depression cohort exhibited significantly elevated stigma scores (SIS) and reduced quality of life (QOL) scores relative to the control group (*p* < 0.001 for both comparisons).

### 3.2 Medication profiles of AECOPD patients

Significant differences in theophyllines use were observed between AECOPD patients with and without depression (*p*<0.05), with a higher prevalence of theophyllines utilization in the depression group. However, no statistically significant differences (*p≥*0.05) were identified between groups in glucocorticoid administration methods or quinolone antibiotic usage (**Table 2**).

**Table 2:** No statistically significant intergroup differences were observed in quinolone utilization rates (***p*** ≥ 0.05) or systemic glucocorticoid administration patterns (*p*≥0.05).

Variable	Category	Control group (n = 156)	Depression group (n = 113)	Z/χ²/t	P-value
Antibiotics	Non-quinolones	127(81.40%)	100(88.50%)	2.497	0.114
	Quinolones	29(18.60%)	13(11.50%)		
Glucocorticoids	Not used	11(7.10%)	8(7.10%)	0.040	1.000
	Inhaled only	25(16.00%)	18(15.90%)		
	Systemic	120(76.90%)	87(77.00%)		
Theophyllines	Not used	37(23.70%)	14(12.40%)	5.474	0.019
	Used	119(76.30%)	99(87.60%)		

The depression group demonstrated significantly higher theophyllines utilization compared to controls (*p* < 0.05).

### 3.3 Laboratory findings in AECOPD patients

Laboratory parameters of all enrolled patients were analyzed. No significant differences were observed between the depression and control groups in routine blood tests, coagulation profiles, or other biochemical markers. Hepatic and renal function tests revealed statistically significant intergroup differences in sodium levels and aspartate aminotransferase (AST) (*p* < 0.05), with the depression group demonstrating elevated calcium ion concentrations and lower AST values compared to controls (**Table 3**).

**Table 3:** The depression group exhibited significantly lower aspartate aminotransferase (AST) levels compared to the control group (*p* < 0.05).

Variable	Control group (n = 156)	Depression group (n = 113)	Z/χ²/t	P-value
White blood cell (WBC, ×10^9^/L)	7.355(5.583~10.525)	7.9(5.91~10.00)	0.530	0.596
Neutrophil percentage (%)	79.300(70.175~85.500)	80(70.95~85.00)	0.501	0.616
Neutrophil count (×10^9^/L)	5.630(4.140~8.590)	5.97(4.225~8.215)	0.414	0.679
Red blood cell (RBC, ×10¹²/L)	4.155 ± 0.672	4.1844 ± 0.642	-0.358	0.720
Hemoglobin (g/L)	125.170 ± 20.055	125.85 ± 19.646	-0.276	0.783
Platelet count (×10^9^/L)	192.530 ± 75.248	186.840 ± 74.251	0.616	0.538
Prothrombin time (PT, sec)	11.300(10.700~12.200)	11.4(10.75~12.15)	0.221	0.825
Activated partial thromboplastin time (APTT, sec)	27.7(25.900~29.975)	28.2(26.050~31.087)	1.484	0.138
Fibrinogen (g/L)	4.105(3.328~5.520)	4.28(3.200~6.105)	0.517	0.605
Thrombin time (TT, sec)	17.600(16.800~18.600)	17.4(16.55~18.50)	-1.014	0.311
D-dimer (μg/mL)	0.670(0.360~1.190)	0.52(0.33~1.32)	-1.235	0.217
Prothrombin activity (%)	87.551 ± 12.394	86.539 ± 12.471	0.659	0.510
Alanine aminotransferase (ALT, U/L)	18.000(13.000~26.750)	17(11~23)	-1.510	0.131
Aspartate aminotransferase (AST, U/L)	23.500(18.250~32.000)	21(17.5~27.5)	-2.103	0.035
Total bilirubin (μmol/L)	12.900(9.000~16.650)	12(8~17)	-0.817	0.414
Urea (mmol/L)	6.850(5.600~8.900)	7.3(5.0~9.3)	-0.430	0.667
Creatinine (μmol/L)	67.000(53.000~89.150)	64(53.45~82.00)	-0.994	0.320
Estimated glomerular filtration rate (eGFR, mL/min/1.73m²)	92.700(74.975~101.175)	93.4(77.6~101.2)	0.522	0.601
Uric acid (mmol/L)	285.000(222.000~382.500)	297(246~385)	0.937	0.349
Glucose (mmol/L)	5.950(4.900~7.800)	5.7(5.05~7.10)	-0.536	0.592
Sodium (mmol/L)	140.050(137.050~141.975)	140.9(137.9~142.9)	2.191	0.028
Calcium (mmol/L)	2.190(2.123~2.270)	2.21(2.135~2.271)	0.654	0.513
Total protein (g/L)	66.721 ± 6.878	65.043 ± 10.212	1.515	0.131
Albumin (g/L)	39.316 ± 3.831	38.638 ± 4.800	1.286	0.199
Potassium (mmol/L)	4.070 ± 0.585	3.9651 ± 0.599	1.431	0.154

Serum sodium concentrations were marginally higher in the depression cohort, though this difference did not reach statistical significance (*p*≥0.05).

### 3.4 Clinical characteristics of AECOPD patients with varying depression severity

Patients with AECOPD and comorbid depression (PHQ-9 score >4) were stratified into mild (86 cases) and moderate-to-severe subgroups (27 cases), with the latter combining moderate and severe categories due to limited sample size in the severe depression subgroup. Comparative analysis revealed significant differences (*p* < 0.05) between subgroups in sex distribution, daily cigarette consumption, and length of hospital stay. The moderate-to-severe depression subgroup exhibited a higher proportion of female patients, prolonged hospitalization, and reduced daily cigarette intake compared to the mild depression group. No significant differences (*p*≥0.05) were observed in age, BMI, educational attainment, comorbidities, mMRC grades, or QOL scores between subgroups (**Table 4**).

**Table 4:** The moderate-to-severe depression subgroup demonstrated: significantly prolonged hospitalization duration, reduced daily cigarette consumption, higher proportion of female patients, compared to the mild depression group (*p* < 0.05 for all comparisons).

Variable	Category	Mild group (n = 85)	Moderate-to-severe group (n = 26)	Z/χ²/t	P-value
Age (years)		76(72~80)	79(71.75~82.50)	0.882	0.378
Length of hospital stay (days)		8(6~9)	10(8.00~13.25)	3.79	<0.001
Hospitalizations within past year (times)		1(1~2)	2(1~3)	1.947	0.052
Sex	Male	64(75.30%)	14(53.80%)	4.384	0.036
	Female	21(24.70%)	12(46.20%)		
BMI		21.425 ± 3.307	20.304 ± 2.865	1.558	0.122
Education level	Primary school or below	74(87.10%)	19(73.10%)	1.928	0.165
	Above primary school	11(12.90%)	7(26.90%)		
Smoking history (years)		30(0~40)	5(0~40)	-1.646	0.100
Comorbidities	No	30(35.30%)	6(23.10%)	1.356	0.244
	Yes	55(64.70%)	20(76.90%)		
Daily cigarette consumption (sticks)		20(0~40)	10(0~20)	-2.232	0.026
mMRCgrade	Grade 0	0(0.00%)	0(0.00%)	0.593	0.939
	Grade 1	4(4.70%)	1(3.80%)		
	Grade 2	14(16.50%)	5(19.20%)		
	Grade 3	18(21.20%)	4(15.40%)		
	Grade 4	49(57.60%)	16(61.50%)		
SIS		52.200 ± 6.886	48.620 ± 9.529	1.781	0.084
QOL		51.0815 ± 3.43923	49.044 ± 4.81678	2.393	0.053

### 3.5 Medication profiles of AECOPD patients with varying depression severity

Significant differences in glucocorticoid dosage were observed between the mild and moderate-to-severe depression subgroups(*p*<0.05), with the moderate-to-severe subgroup demonstrating higher glucocorticoid utilization. No statistically significant differences (*p*≥0.05) were identified between subgroups in quinolone antibiotic or theophyllines administration patterns (**Table 5**).

**Table 5:** Significant intergroup disparities were observed in glucocorticoid administration methods (*p* < 0.05).

Variable	Category	Mild group (n = 85)	Moderate-to-severe group (n = 26)	Z/χ²/t	P-value
Antibiotics	Non-quinolones	76(89.40%)	22(84.60%)	0.101	0.751
	Quinolones	9(10.60%)	4(15.40%)		
Glucocorticoids	Not used	68(80.00%)	17(65.40%)	6.353	0.037
	Inhaled only	3(3.50%)	5(19.20%)		
	Systemic	14(16.50%)	4(15.40%)		
Theophyllines	Not used	13(15.03%)	1(3.80%)	1.443	0.230
	Used	72(84.70%)	25(96.20%)		

The moderate-to-severe depression subgroup exhibited a significantly higher inhaled glucocorticoid utilization rate compared to the mild depression subgroup.

### 3.6 Laboratory findings in AECOPD patients with varying depression severity

Laboratory investigations including routine blood tests, coagulation profiles, and hepatic/renal function assessments were performed across depression severity subgroups. Analytical results demonstrated marginally lower aspartate aminotransferase (AST) levels and calcium ion concentrations in the mild depression subgroup compared to the moderate-to-severe subgroup. However, no statistically significant differences (*p*>0.05) were observed between subgroups in routine blood parameters, coagulation indices, renal function markers, or other hepatic/electrolyte profiles (**Table 6**).

**Table 6:** The moderate-to-severe depression subgroup exhibited significantly higher aspartate aminotransferase (AST) levels compared to the mild depression subgroup (*p* < 0.05).

Variable	Mild group (n = 85)	Moderate-to-severe group (n = 26)	Z/χ²/t	P-value
White blood cell (WBC, ×10^9^/L)	8.030(5.880~10.030)	7.415(6.078~10.553)	0.181	0.856
Neutrophil percentage (%)	79.900(70.900~84.450)	81.600(73.675~89.000)	1.267	0.205
Neutrophil count (×10^9^/L)	5.970(4.225~8.215)	6.185(4.103~9.603)	0.338	0.736
Red blood cell (RBC, ×10¹²/L)	4.208 ± 0.682	4.094 ± 0.580	0.770	0.443
Hemoglobin (g/L)	126.700 ± 20.657	123.000 ± 19.127	0.812	0.419
Platelet count (×10^9^/L)	191.310 ± 71.297	170.310 ± 67.690	1.330	0.186
Prothrombin time (PT, sec)	11.218 ± 2.112	11.769 ± 1.132	-1.273	0.206
Activated partial thromboplastin time (APTT, sec)	28.000(25.850~31.050)	27.150(25.725~29.975)	-0.951	0.342
Fibrinogen (g/L)	4.142(3.260~5.940)	3.620(2.868~6.373)	-0.501	0.616
Thrombin time (TT, sec)	17.700(16.600~18.500)	17.350(16.750~19.425)	0.324	0.746
D-dimer (μg/mL)	0.500(0.335~1.345)	0.610(0.328~1.073)	-0.052	0.958
Prothrombin activity (%)	88.368 ± 15.132	83.031 ± 11.737	1.651	0.102
Alanine aminotransferase (ALT, U/L)	16.000(11.000~22.500)	20.000(12.750~26.250)	1.262	0.207
Aspartate aminotransferase (AST, U/L)	20.000(17.000~24.500)	24.000(18.750~35.250)	2.399	0.016
Total bilirubin (μmol/L)	12.000(9.000~16.500)	13.550(7.000~17.050)	0.150	0.881
Urea (mmol/L)	7.057 ± 2.501	7.946 ± 2.890	-1.529	0.129
Creatinine (μmol/L)	61.000(52.050~78.000)	71.000(58.250~93.000)	1.769	0.077
Estimated glomerular filtration rate (eGFR, mL/min/1.73m²)	89.544 ± 22.128	83.701 ± 21.328	1.188	0.237
Uric acid (mmol/L)	303.940 ± 102.200	351.810 ± 129.480	-1.958	0.053
Glucose (mmol/L)	5.900(5.050~6.988)	5.600(5.075~7.325)	0.014	0.989
Sodium (mmol/L)	140.442 ± 3.581	140.165 ± 3.626	0.344	0.731
Calcium (mmol/L)	2.192 ± 0.119	2.254 ± 0.172	-2.077	0.040
Total protein (g/L)	63.000(59.600~71.500)	64.850(59.975~69.250)	0.884	0.377
Albumin (g/L)	38.431 ± 4.852	38.962 ± 4.715	-0.491	0.624
Potassium (mmol/L)	3.962 ± 0.598	3.924 ± 0.676	0.272	0.786

Serum calcium concentrations were marginally elevated in the moderate-to-severe subgroup, though this difference did not reach statistical significance (*p*≥0.05).

## 4 Discussion

This study found a high depression prevalence (42.01%) in AECOPD patients. This rate is 1.6-fold higher than the 26% pooled estimate from a meta-analysis of 41 COPD studies (15) and exceeds the 31% reported in a recent Beijing inpatient cohort (16). Female sex, long smoking history, clinical severity (mMRC), stigma (SIS scores), reduced QOL, and theophylline use were significantly associated with depression. These associations echo the prospective data of Huang et al. (17), who showed that baseline PHQ-9≥10 doubles the risk of subsequent exacerbations. Severity subgroup analyses revealed heterogeneity in sex distribution, glucocorticoid use, daily cigarette consumption, and hospitalization duration. The 2-day increase in median stay aligns with the +1.3-day prolongation reported by Blakemore et al. (18) in depressed COPD patients managed in primary care. The management of more complex cases, particularly treatment-resistant depression (TRD), is recognized as a clinical priority in the GOLD 2025 strategy document (19). This prevalence is higher than in the general population and may be linked to acute physiological stress (e.g., hypoxia, inflammation) and psychological burden (e.g., isolation, disease fear). The management of more complex cases, particularly treatment-resistant depression (TRD) — defined as an inadequate response to at least two trials of antidepressant therapy — poses a significant clinical challenge, as highlighted in recent national consensus guidelines and epidemiological reviews (20, 21).

Female sex as a risk factor aligns with prior research (22). Explanations include: Socioeconomic Burden: Recurrent AECOPD costs and COPD disability disproportionately affect women. Accelerated Physical Decline: Faster age-related decline may lead to earlier workforce loss and helplessness. Seasonal Synchronicity: AECOPD peaks in autumn/winter coincide with seasonal affective disorder (23), potentially amplifying symptoms in women. Symptom Sensitivity: Women show greater symptom awareness (24), worsening anxiety during exacerbations. Social Role Stress: Disease progression impeding multifaceted societal roles increases distress. Hormonal Fluctuations: Hormonal variations (menstrual cycle, menopause) may increase depression vulnerability (25, 26).

A significant link between prolonged smoking and depression highlights complex interactions. Nicotine dependence may dysregulate dopaminergic reward pathways (27, 28), inducing transient pleasure but elevating depression risk chronically. Smoking also induces systemic inflammation (29) (e.g., IL-6, TNF-α), potentially disrupting neurotransmitter balance via the gut-lung-brain axis (30), worsening mood.

Theophylline use correlated with depression risk (31). While effective for ventilation, its CNS stimulant properties (32) may contribute to neuropsychiatric issues. Phosphodiesterase inhibition increases cAMP for bronchodilation but may concurrently cause CNS hyperexcitability and neurotransmitter imbalance, increasing depression susceptibility (33). For patients exhibiting treatment resistance, augmentation strategies with agents acting on different neurotransmitter systems, such as cariprazine (a D3/D2 and serotonergic modulator), have shown promise in other treatment-resistant affective disorders (34) and could represent a novel therapeutic avenue worthy of exploration in this comorbid population.

Subgroup analyses showed two inverse associations in moderate-to-severe depression: reduced daily cigarette consumption and longer hospitalization. This paradox may reflect complex pathophysiology. Smoking reduction aligns with the Illness Perception-Behavior Modification Model (35), where patients reduce smoking recognizing symptom synergy. However, nicotine withdrawal symptoms (e.g., anxiety) (36) may worsen depression (37), creating a vicious cycle.

The glucocorticoid use disparity is notable. While effective for inflammation and respiratory function, their neuropsychiatric side effects (e.g., mood instability) are documented, especially with high-dose/long regimens (38). This necessitates cautious optimization in patients with moderate-to-severe depression. Future studies should balance anti-inflammatory efficacy with psychiatric safety.

This study first reveals in AECOPD patients the significant predictive roles of perceived stigma (SIS) and QOL in depression. Heightened stigma may reduce care-seeking (39), compromising treatment. Diminished QOL reflects physical and psychosocial impairment. Integrating psychological interventions (e.g., cognitive behavioral therapy, stigma reduction) into AECOPD management is crucial to disrupt the somatic-psychological distress cycle. The potential of CBT-based digital tools to support the embodied reintegration following pharmacologically induced plasticity, as suggested in esketamine research (40), points towards a truly multimodal and personalized approach to care.

While overlaps exist with prior AECOPD insomnia research (e.g., female sex, severity), depression shows stronger links to psychosocial factors (stigma, QOL) and specific pharmacotherapy (theophyllines), whereas sleep disturbances correlate more with nocturnal symptoms (13). This divergence suggests tailored approaches: prioritize psychosocial support for depression and optimized symptom control/oxygen therapy for sleep.

This study has several limitations. Although the total sample size (n=269) was sufficient for primary comparisons, the subgroup with severe depression (n=3) was too small for reliable analysis, necessitating cautious interpretation of these results. The single-center design may limit generalizability, and residual confounding—particularly by unmeasured socioeconomic factors—cannot be excluded. Furthermore, the cross-sectional design prevents causal inference. Future large-scale prospective studies are needed for validation.

In summary, we identified multiple depression risk factors in AECOPD: female sex, smoking history, theophylline use, disease severity, stigma, and impaired QOL. Severity subgroup analyses linked sex, glucocorticoid use, daily cigarette consumption, and hospitalization duration to aggravated depression. To validate these findings, our team plans multicenter studies with expanded samples and follow-up to evaluate characteristics and identify precise interventions, optimizing clinical practice. The insights from recent research on TRD management (20, 21), novel pharmacological mechanisms (34), and the interplay between neuropharmacological action and psychological change (40) provide valuable frameworks for developing these future interventions.

## 5 Conclusions

This study demonstrates that patients with AECOPD who are female, receive theophylline therapy, have more severe disease, report higher levels of perceived stigma, or have poorer quality of life are at a significantly increased risk of developing depression. Furthermore, depression severity is associated with female sex, prolonged hospitalization, and glucocorticoid use. However, it should be noted that certain subgroup analyses may be limited by small sample sizes, and these findings should be interpreted with caution until validated in larger cohorts. Optimizing AECOPD treatment to alleviate clinical symptoms may help reduce the incidence of comorbid depression. These results highlight the importance of incorporating psychosocial assessments and tailored interventions into standard COPD care to address the combined burden of respiratory and depressive illness.

## Data Availability

The raw data supporting the conclusions of this article will be made available by the authors, without undue reservation.
