# Correction: Impact of comorbid depression on quality of life and disease progression in chronic obstructive pulmonary disease: a correlational analysis

**DOI:** 10.3389/fpsyt.2025.1725540

**Published:** 2025-10-28

**Authors:** Yu Chen, Qianqian Gao, Hongqin Jia, Kang Qian, Hongbin Zhu

**Affiliations:** ^1^ The Fourth Affiliated Hospital of Anhui Medical University, Chaohu, China; ^2^ Department of Respiratory and Critical Care Medicine, Fuyang People’s Hospital, Fuyang, China; ^3^ Department of Respiratory and Critical Care Medicine, The Fourth Affiliate Hospital of Anhui Medical University, Chaohu, China

**Keywords:** AECOPD, depression, risk factors, quality of life, stigma

Author “Qianqian Gao” was erroneously omitted as equal contributing author.

The original version of this article has been updated.

